# Characteristics, Prognosis, and Prediction Model of Heart Failure Patients in Intensive Care Units Based on Preserved, Mildly Reduced, and Reduced Ejection Fraction

**DOI:** 10.31083/j.rcm2406165

**Published:** 2023-06-06

**Authors:** Fang Tao, Wenguang Wang, Hongmei Yang, Xiaoyu Han, Xun Wang, Yuhan Dai, Aihong Zhu, Yue Han, Pan Guo

**Affiliations:** ^1^Medical Department, Qinhuangdao First Hospital, 066000 Qinhuangdao, Hebei, China; ^2^Department of Cardiology, Qinhuangdao First Hospital, 066000 Qinhuangdao, Hebei, China

**Keywords:** heart failure, ejection fraction, ICU, survival, prediction model, follow-up

## Abstract

**Background:**

Heart failure (HF) patients in intensive care units (ICUs) are rather poorly studied based on varying left ventricular ejection fraction (LVEF) classification. Characteristics and prognosis of patients in ICUs with HF with mildly reduced ejection fraction (HFmrEF), HF with reduced ejection fraction (HFrEF) and HF with preserved ejection fraction (HFpEF) require further clarification.

**Methods:**

Data involving clinical information and 4-year follow-up records of HF patients were extracted and integrated from the Medical Information Mart for Intensive Care III (MIMIC-III) database. Tests were carried out to identity differences among these three HF subtypes. Prognostic analyses were performed using Kaplan-Meier survival analysis and Cox proportional-hazards regression modeling. To develop a novel prediction nomogram, forward selection was used as the best-fit model. Prognostic heterogeneity of the subgroups prespecified by stratification factors in pairwise comparisons was presented using forest plots.

**Results:**

A total of 4150 patients were enrolled in this study. HFmrEF had the lowest all-cause mortality rate during the 4-year follow-up, which was significantly different from HFrEF and HFpEF (Log-Rank *p <* 0.001). The Cox proportional-hazards regression model also showed that a comparison of HFrEF versus HFmrEF indicated a hazard ratio (HR) of 0.76 (95% CI 0.61–0.94, *p* = 0.011) and HFrEF versus HFpEF indicated a HR 0.93 (95% CI 0.82–1.07, *p *= 0.307). Following a multivariable analysis, 13 factors were confirmed as independent. A new nomogram was established and quantified with a concordance index (C-index) of 0.70 (95% CI 0.67–0.73), and the internal validation indicated the accuracy of the model. Stratification factors such as a history of coronary artery bypass grafting (CABG) and comorbidity of chronic obstructive pulmonary disease (COPD) induced prognostic heterogeneity among the three subtypes.

**Conclusions:**

Clinical characteristics and prognosis significantly varied among the three subtypes of HF patients in ICUs, with HFmrEF patients achieving the best prognosis. The novel prediction model, tailored for this population, showed a satisfying prediction ability.

## 1. Introduction

Heart failure (HF) remains one of the leading causes of death and is increasing in incidence [1]. Five-year mortality rates have increased from 53% to 67% [2]. Moreover, HF is a common diagnosis for patients in the intensive care unit (ICU) and 20% of hospitalized HF patients in the USA were admitted to the ICU [3].

Recently, guidelines have introduced many new interpretations to the three principal subtypes of HF, these are HF with preserved ejection fraction (HFpEF), HF with mildly reduced ejection fraction (HFmrEF), and HF with reduced ejection fraction (HFrEF) [1]. Literature regarding characteristics, mechanisms, and prognosis of these subtypes has underlined their differences and classification [4]. However, results of published articles vary greatly, and many of them are limited to new onset or acute HF patients. Additionally, patients with HF in ICUs remain rather poorly studied. Therefore, it is of importance to better understand this unique patient population. Furthermore, a new prognostic model, specifically designed for HF patients in ICU, was developed to predict the risk of mortality in this patient population.

## 2. Materials and Methods

### 2.1 Database Source

Medical Information Mart for Intensive Care III (MIMIC-III) [5] is a large, single-center database containing over sixty thousand patients spanning from 2001 to 2012. This database contains anonymous, comprehensive clinical data from patients admitted to the Beth Israel Deaconess Medical Center, and it is open to international researchers. MIMIC-III includes patient vital signs, medications, laboratory findings, nursing records and observations, fluid intake and output, procedure and diagnostic codes, imaging results, and patient survival data. We collected data from MIMIC-III database for this study; however, patient consent and ethic approvals were not necessary for this investigation.

### 2.2 Study Population

Patients diagnosed with HF and over 18 years of age were enrolled in this study. Exclusion criteria were: (1) Patients with incomplete data of left ventricular ejection fraction (LVEF); (2) Patients who died in the hospital or within 24 hours after discharge.

We only extracted data of the first ICU admission during the initial hospitalization if patients had multiple records of hospitalization or multiple ICU admissions during a same hospitalization. According to 2021 European Society of Cardiology (ESC) heart failure guidelines [1], patients were divided into three HF-groups: HFrEF (LVEF ≤40%), HFmrEF (LVEF 41–49%), and HFpEF (LVEF ≥50%).

### 2.3 Data Extraction

Data were extracted by PostgreSQL 9.6 software and SQL (Berkeley, California, USA). Among the data extracted were demographics, ICU stay time, ICU type, complication, laboratory and imaging examination, treatment, and time of death. The formula (2 × ${\mathrm{Na}}^{+}$ + $K^{+}$) + (glucose/18) + (urea/2.8) was used to calculate plasma osmotic pressure (POP). The Cockcroft-Gault-Glomerular filtration rate (CG-GFR) was measured using the formula “Male: (140 – age) × weight (kg) × 1.23/creatinine (μmol/L); Female: (140 – age) × weight (kg) × 1.03/creatinine (μmol/L)” was used to calculate GFR. For laboratory values, we generally extracted the initial value in each index. The minimum value of hemoglobin (HB min), the maximum value of $K^{+}$ (K max), the minimum value of $K^{+}$ (K min), and the maximum value of white blood cell (WBC max) were extracted since these values may contribute significantly to impacts on prognosis.

### 2.4 Endpoints

Since the MIMIC-III database was issued in 2016 with the last patient enrollment in 2012, we chose 4 years as the observation time, and all-cause mortality was chosen as the endpoint. MIMIC-III confirmed and collected information of all-cause mortality from the Social Security Administration Death Master File; thus, no patient was lost to follow-up.

### 2.5 Statistical Analysis

The Kolmogorov-Smirnov test was used to determine whether continuous variables fit a normal distribution. Continuous variables were expressed as the mean ± the standard deviation if data followed a normal distribution. The F test was used to analyze homogeneity of variance among HF groups. Differences among groups were analyzed by Student *t*-test if the data satisfied variance homogeneity tests, or the Satterthwaite *t*-test if not. Continuous variables disqualified from normal distribution were represented by the median and interquartile range (IQR) M (P25,75). Comparisons of two groups were made using the Mann–Whitney U test, and for comparisons of three groups, the Kruskal-Wallis test was used. Enumeration data were described by number (n) and percentage (%). The Pearson’s Chi-square test was applied to verify the difference among HF groups. Prognosis was estimated using the Kaplan-Meier survival method, and the HF group difference was compared using the log-rank test. The Cox proportional-hazards regression model was used for both univariate and multivariate survival analyses. The spline function was employed to test whether there was a linear relationship between continuous variables and prognosis. If a linear relationship was detected the continuous variables were directly analyzed. If not, variables were converted into categorical variables by dividing them into three groups of tertiles, or their upper and lower limits of normal values, to facilitate comparison between groups.

All variables were first analyzed using the univariate Cox model, and then the significant variables were further included in a multivariate Cox analysis to confirm independent factors. All independent factors were filtered through multiple regression steps to formulate a prognostic model. After testing the three directions, specifically, forward, backward, and stepwise, forward selection was selected as the direction providing the best fit [6]. Predictive performance of the model was quantified by determining the concordance index (C-index), and internal validation of 1000 bootstrap resamples were determined by calibration curves and bootstrap-corrected C-index.

To examine the heterogeneity of prognosis among the three HF groups, exploratory analyses were performed across prespecified subgroups that were defined according to stratification factors obtained by forest plots.

A two-tailed *p <* 0.05 was considered statistically significant. Analyses were performed using R software (version 4.1.1, R Foundation for Statistical Computing, Vienna, Austria) and SPSS 24.0 (IBM Corp., Armonk, NY, USA).

## 3. Results

### 3.1 Patient Characteristics

A total of 4150 patients were recruited in this study (**Supplementary Fig. 1**). The proportion of the three HF-groups within this study group were HFrEF n = 1234 (29.73%), HFmrEF n = 312 (7.50%), and HFpEF n = 2604 (62.75%). Characteristics varied in many aspects among these three HF groups and details of demographic characteristics, ICU stay time, comorbidities, laboratory tests. imaging results, treatments, and main diagnoses are shown in Tables 1,2.

**Table 1.** **Baseline of three groups of heart failure**.

		HFrEF	HFmrEF	HFpEF	Overall	HFrEF *vs.* HFmrEF	HFmrEF *vs.* HFpEF
		(n = 1234, 29.73%)	(n = 312, 7.50%)	(n = 2604, 62.75%)	*p*	*p*	*p*
Age (years)		71.10 (61.24–81.15)	73.58 (63.72–81.49)	75.23 (64.10–83.37)	<0.001	0.147	0.033
Weight (kg)		79.50 (67.65–92.72)	80.90 (67.85–97.00)	78.30 (65.67–95.00)	0.152	0.104	0.061
Male		828 (67.10%)	205 (65.71%)	1112 (42.70%)	<0.001	0.640	<0.001
ICU stay time (days)		3.10 (1.68–5.47)	2.79 (1.52–5.00)	2.93 (1.66–5.66)	0.297	0.126	0.144
Ethnicity							
	White	937 (75.93%)	247 (79.17%)	1975 (75.84%)	0.422	0.228	0.193
	Black/African American	103 (8.35%)	19 (6.09%)	222 (8.53%)	0.336	0.186	0.140
	Hispanic or Latino	38 (3.08%)	7 (2.24%)	58 (2.23%)	0.274	0.433	0.985
	Asian	26 (2.11%)	5 (1.60%)	51 (1.96%)	0.844	0.570	0.665
	Unable to obtain	104 (8.43%)	26 (8.33%)	231 (8.87%)	0.876	0.957	0.752
	Other	26 (2.11%)	8 (2.56%)	67 (2.57%)	0.674	0.623	0.993
ICU type							
	CCU	509 (41.25%)	107 (34.29%)	543 (20.85%)	<0.001	0.025	<0.001
	CSRU	370 (29.98%)	117 (37.50%)	554 (21.27%)	<0.001	0.011	<0.001
	MICU	258 (20.91%)	63 (20.19%)	1094 (42.01%)	<0.001	0.781	<0.001
	SICU	70 (5.67%)	16 (5.13%)	259 (9.95%)	<0.001	0.708	0.006
	TSICU	27 (2.19%)	9 (2.88%)	154 (5.91%)	<0.001	0.466	0.028
SOFA		4.00 (2.00–7.00)	5.00 (3.00–7.00)	4.00 (2.00–6.00)	0.005	0.243	0.007
AF		588 (47.65%)	170 (54.49%)	1325 (50.88%)	0.050	0.031	0.009
AMI		132 (10.70%)	34 (10.90%)	94 (3.61%)	<0.001	0.919	<0.001
MI		344 (27.88%)	66 (21.15%)	269 (10.33%)	<0.001	0.016	<0.001
Coronary disease		782 (63.40%)	210 (67.31%)	852 (32.72%)	<0.001	0.195	<0.001
COPD		34 (2.76%)	7 (2.24%)	145 (5.57%)	<0.001	0.615	0.013
Hypertension		535 (43.35%)	157 (50.32%)	1126 (43.24%)	0.055	0.027	0.017
Diabetes		492 (39.87%)	121 (38.78%)	921 (35.37%)	0.021	0.726	0.234
CABG		284 (23.01%)	86 (27.56%)	267 (10.25%)	<0.001	0.092	<0.001
PCI		172 (13.94%)	41 (13.14%)	124 (4.76%)	<0.001	0.715	<0.001
Main diagnoses							
	MI	332 (26.90%)	103 (33.02%)	313 (12.02%)	<0.001	0.032	<0.001
	HF	331 (26.82%)	39 (12.50%)	251 (9.64%)	<0.001	<0.001	0.012
	Cardiacvalve disease	111 (9.00%)	52 (16.67%)	300 (11.52%)	<0.001	<0.001	0.008
	Septicemia	51 (4.12%)	18 (5.77%)	218 (8.37%)	<0.001	0.211	0.111
	Hemorrhage	33 (2.67%)	13 (4.17%)	145 (5.57%)	<0.001	0.166	0.301
	Respiratory failure	25 (2.02%)	9 (2.88%)	140 (5.38%)	<0.001	0.355	0.059
	Pneumonia	13 (1.05%)	5 (1.60%)	82 (3.15%)	<0.001	0.083	0.129
	Acute kidney failure	13 (1.05%)	3 (0.96%)	56 (2.15%)	0.028	0.588	0.159
	Others	325 (22.34%)	70 (22.44%)	1099 (42.20%)	<0.001	0.158	<0.001

HFrEF, heart failure with reduced ejection fraction; HFmrEF, heart failure with mildly reduced ejection fraction; HFpEF, heart failure with preserved ejection fraction; *vs.*, versus; kg, kilogram; ICU, intensive care unit; CCU, coronary care unit; CSRU, cardiovascular surgery rehabilitation unit. MICU, medical intensive care unit; SICU, surgery intensive care unit; TSICU, trauma and surgical intensive care unit; SOFA, sepsis-related organ failure assessment; AF, atrial fibrillation; AMI, acute myocardial infarction; MI, myocardial infarction; COPD, chronic obstructive pulmonary disease; CABG, coronary artery bypass grafting; PCI, percutaneous coronary intervention.

**Table 2.** **Treatments and examination results**.

	HFrEF	HFmrEF	HFpEF	Overall	HFrEF *vs.* HFmrEF	HFmrEF *vs.* HFpEF
	(n = 1234, 29.73%)	(n = 312, 7.50%)	(n = 2604, 62.75%)	*p*	*p*	*p*
ACEI/ARB	894 (72.45%)	195 (62.50%)	1250 (48.00%)	<0.001	<0.001	<0.001
β-blocker	1036 (94.65%)	286 (91.67%)	2077 (79.76%)	<0.001	<0.001	<0.001
Loop diuretics	1090 (88.33%)	266 (85.26%)	2203 (84.60%)	0.008	0.140	0.761
Spironolactone	153 (12.40%)	12 (3.85%)	142 (5.45%)	<0.001	<0.001	0.230
Statin	975 (79.01%)	245 (78.53%)	1592 (61.14%)	<0.001	0.850	<0.001
Anticoagulant	542 (43.92%)	128 (41.03%)	986 (37.86%)	0.002	0.356	0.278
Aspirin	1092 (88.49%)	271 (86.86%)	1721 (66.09%)	<0.001	0.425	<0.001
Anti-ADP	411 (33.31%)	111 (35.58%)	426 (16.36%)	<0.001	0.449	<0.001
Digitalis	165 (13.37%)	23 (7.37%)	196 (7.53%)	<0.001	0.004	0.922
SCr (mg/dL)	1.10 (0.90–1.60)	1.10 (0.80–1.48)	1.10 (0.80–1.60)	0.002	0.028	0.899
GFR (mL/min)	60.34 (37.14–91.41)	65.71 (39.33–99.30)	56.18 (34.84–89.76)	0.008	0.066	0.005
HB (g/dL)	10.80 (9.30–12.40)	10.40 (9.00–11.90)	10.30 (9.10–11.70)	<0.001	0.004	0.615
HB min (g/dL)	9.10 (8.10–10.60)	8.90 (8.00–10.10)	8.80 (7.90–9.90)	<0.001	0.033	0.254
Plt (${\mathrm{10}}^{9}$/L)	201.00 (154.00–270.00)	182.50 (139.50–242.50)	202.00 (146.00–267.00)	0.001	<0.001	0.001
K (mmol/L)	4.30 (3.90–4.80)	4.20 (3.90–4.80)	4.10 (3.70–4.60)	<0.001	0.917	0.001
K max (mmol/L)	5.00 (4.60–5.50)	5.00 (4.50–5.50)	4.80 (4.50–5.33)	<0.001	0.571	0.013
K min (mmol/L)	3.50 (3.20–3.70)	3.50 (3.20–3.70)	3.40 (3.10–3.70)	<0.001	0.920	0.004
Na (mmol/L)	137.00 (135.00–140.00)	137.00 (135.00–140.00)	138.00 (136.00–141.00)	<0.001	0.469	<0.001
Urea (mg/dL)	24.00 (17.00–39.00)	21.00 (15.25–33.00)	23.00 (16.00–38.00)	0.017	0.004	0.059
WBC (${\mathrm{10}}^{9}$/L)	10.90 (8.00–14.93)	11.45 (8.53–14.30)	10.90 (7.80–14.80)	0.312	0.389	0.171
WBC max (${\mathrm{10}}^{9}$/L)	13.95 (10.20–18.60)	14.40 (11.00–18.50)	14.30 (10.60–19.30)	0.109	0.219	0.948
POP (mmol/L)	297.11 (290.80–304.62)	295.38 (289.54–302.82)	298.56 (291.59–306.30)	<0.001	0.007	<0.001
LVEF (%)	30.00 (22.50–35.00)	45.00 (42.50–47.50)	55.00 (55.00–55.00)	<0.001	<0.001	<0.001

ACEI/ARB, angiotensin-converting enzyme inhibitors/angiotensin receptor blocker; ADP, adenosine diphosphate; SCr, serum creatinine; GFR, Glomerular filtration rate; HB, hemoglobin; Plt, platelet; K, potassium ion; Na, sodium ion; Urea, urea nitrogen; WBC, white blood cell; POP, plasma osmotic pressure; LVEF, left ventricular ejection fraction.

### 3.2 Prognosis and Prognostic Factors

**Fig. 1.**
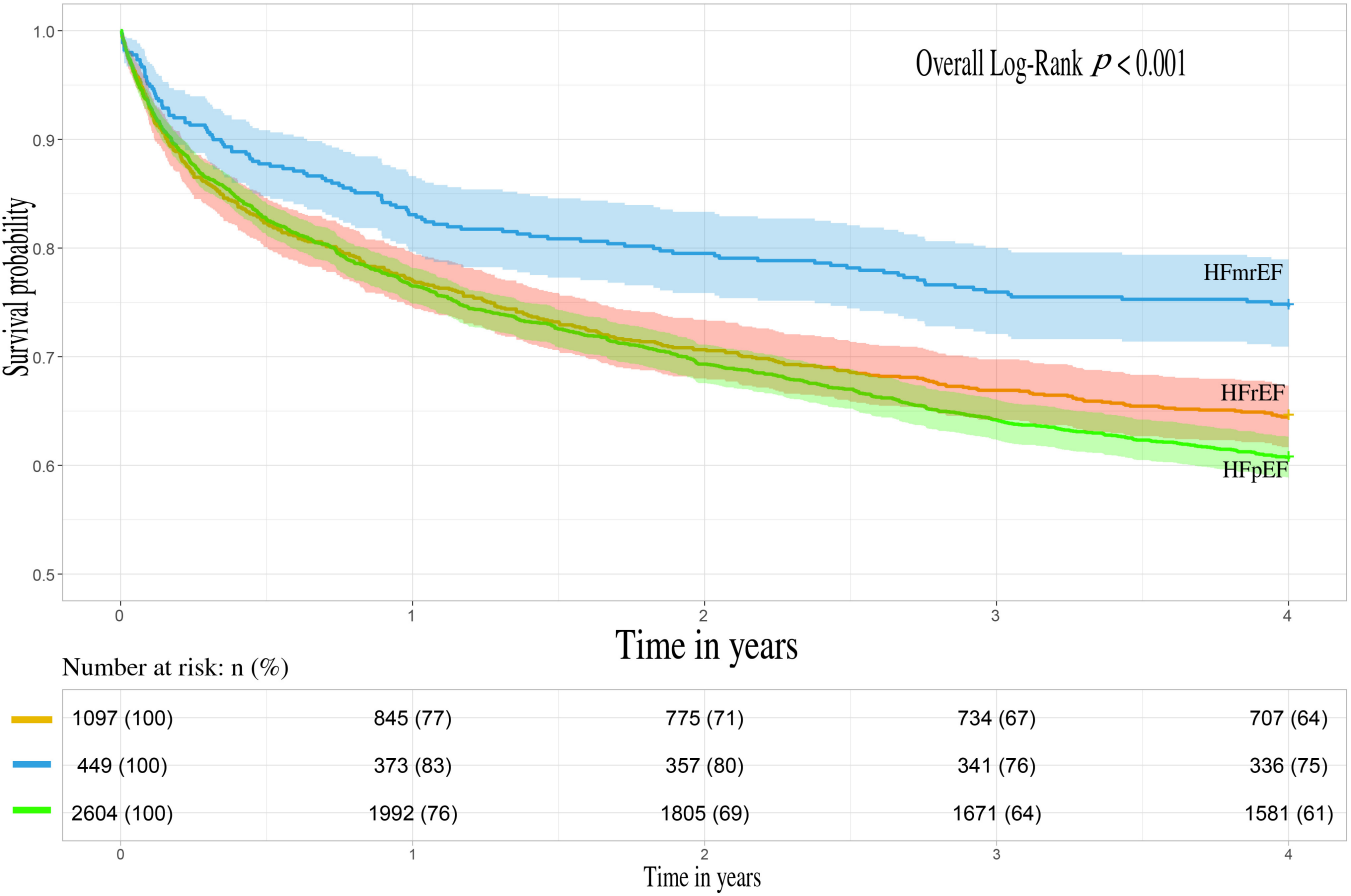
**Kaplan–Meier survival analysis depicted cumulative survival of heart failure patients during 4-year post-discharge**. Heart failure with reduced ejection fraction (HFrEF), heart failure with mildly reduced ejection fraction (HFmrEF), heart failure with preserved ejection fraction (HFpEF).

Significant differences were observed in the all-cause mortality rates among the three HF-groups (overall Log-Rank *p <* 0.001). HFmrEF patients displayed the lowest mortality rate during the four-year follow-up period. HFpEF patients showed a similar mortality rate with HFrEF patients in the first two years of follow-up, however, the rate was higher in the subsequent two years (**Supplementary Table 1** and Fig. 1). The Cox proportional hazards model also showed that the assigned HF group significantly influenced patient survival. Taking HFrEF as a reference, univariate analysis suggested HFmrEF as favorable (HR 0.68, 95% CI 0.53–0.86, *p = *0.002) while those in the HFpEF group was not favorable (HR 1.16, 95% CI 1.04–1.30, *p = *0.010) (**Supplementary Table 2**). Using multivariate analysis, HFmrEF (HR 0.76, 95% CI 0.61–0.94, *p = *0.011) and HFpEF (HR 0.93, 95% CI 0.82–1.07, *p = *0.307) both showed favorability, although no significance was observed in HFpEF. Additionally, age, weight, gender, ICU stay time, ICU type, atrial fibrillation (AF), chronic obstructive pulmonary disease (COPD), hypertension, coronary artery bypass grafting (CABG), GFR, main diagnoses and HB min were also confirmed as independent influence factors using univariate analysis (**Supplementary Table 2**) and further multivariate adjustment (Table 3). Table 3 details the prognostic impacts of different subgroups in each factor.

**Table 3.** **Multivariable Cox proportional-hazards regression model for all-cause mortality**.

Factors		HR (95% CI)	*p*
HF-group			
	HFrEF	REF	REF
	HFmrEF	0.76 (0.61, 0.94)	0.011
	HFpEF	0.93 (0.82, 1.07)	0.307
Age (per 1 year)		1.03 (1.03, 1.04)	<0.001
Weight (kg)			
	<70	REF	REF
	70–87.8	0.84 (0.74, 0.95)	0.006
	>87.8	0.68 (0.59, 0.79)	<0.001
Gender			
	Female	REF	REF
	Male	1.21 (1.09, 1.36)	0.001
ICU stay time (days)			
	<2.1	REF	REF
	2.1–4.8	1.04 (0.91, 1.18)	0.600
	>4.8	1.30 (1.13, 1.50)	<0.001
ICU type			
	CCU	REF	REF
	CSRU	0.90 (0.73, 1.11)	0.326
	MICU	1.25 (1.08, 1.44)	0.003
	SICU	1.18 (0.96, 1.45)	0.109
	TSICU	1.16 (0.91, 1.42)	0.224
SOFA			
	0–3	REF	REF
	3–6	1.12 (0.99, 1.26)	0.078
	>6	1.11 (0.96, 1.29)	0.149
AMI		0.74 (0.55, 1.01)	0.057
MI		0.93 (0.77, 1.11)	0.399
AF		1.24 (1.11, 1.38)	<0.001
Coronary disease		1.04 (0.91, 1.18)	0.570
COPD		1.55 (1.26, 1.91)	<0.001
Hypertension		0.72 (0.65, 0.81)	<0.001
Diabetes		0.98 (0.88, 1.10)	0.714
CABG		0.47 (0.37, 0.61)	<0.001
ACEI/ARB		0.92 (0.83, 1.03)	0.151
β-blocker		0.93 (0.80, 1.08)	0.328
Spironolactone		1.33 (1.10, 1.60)	0.003
Statin		0.97 (0.86, 1.10)	0.657
Digitalis		1.03 (0.87, 1.22)	0.729
Aspirin		0.98 (0.86, 1.17)	0.752
GFR (mL/min)			
	<80	REF	REF
	80–120	0.79 (0.66, 0.93)	0.006
	>120	0.80 (0.63, 1.02)	0.076
HB min (g/dL)			
	<11	REF	REF
	11–16	0.74 (0.62, 0.87)	<0.001
	>16	1.19 (0.17, 8.36)	0.861
K min (mmol/L)			
	<3.5	REF	REF
	3.5–5.5	0.97 (0.86, 1.08)	0.569
	>5.5	None	
POP (mmol/L)			
	<280	REF	REF
	280–320	0.93 (0.71, 1.21)	0.581
	>320	1.01 (0.74, 1.40)	0.930
Main diagnoses			
	MI	REF	REF
	HF	0.97 (0.78, 1.20)	0.779
	Cardiacvalve disease	0.349 (0.26, 0.47)	<0.001
	Septicemia	0.74 (0.57, 0.97)	0.031
	Hemorrhage	0.84 (0.63, 1.12)	0.241
	Respiratory failure	0.73 (0.53, 0.99)	0.041
	Pneumonia	0.68 (0.48, 0.97)	0.036
	Acute kidney failure	0.89 (0.62, 1.30)	0.556
	Others	0.88 (0.72, 1.08)	0.234

HR, hazard ratio; REF, reference group.

### 3.3 Prognostic Model

**Fig. 2.**
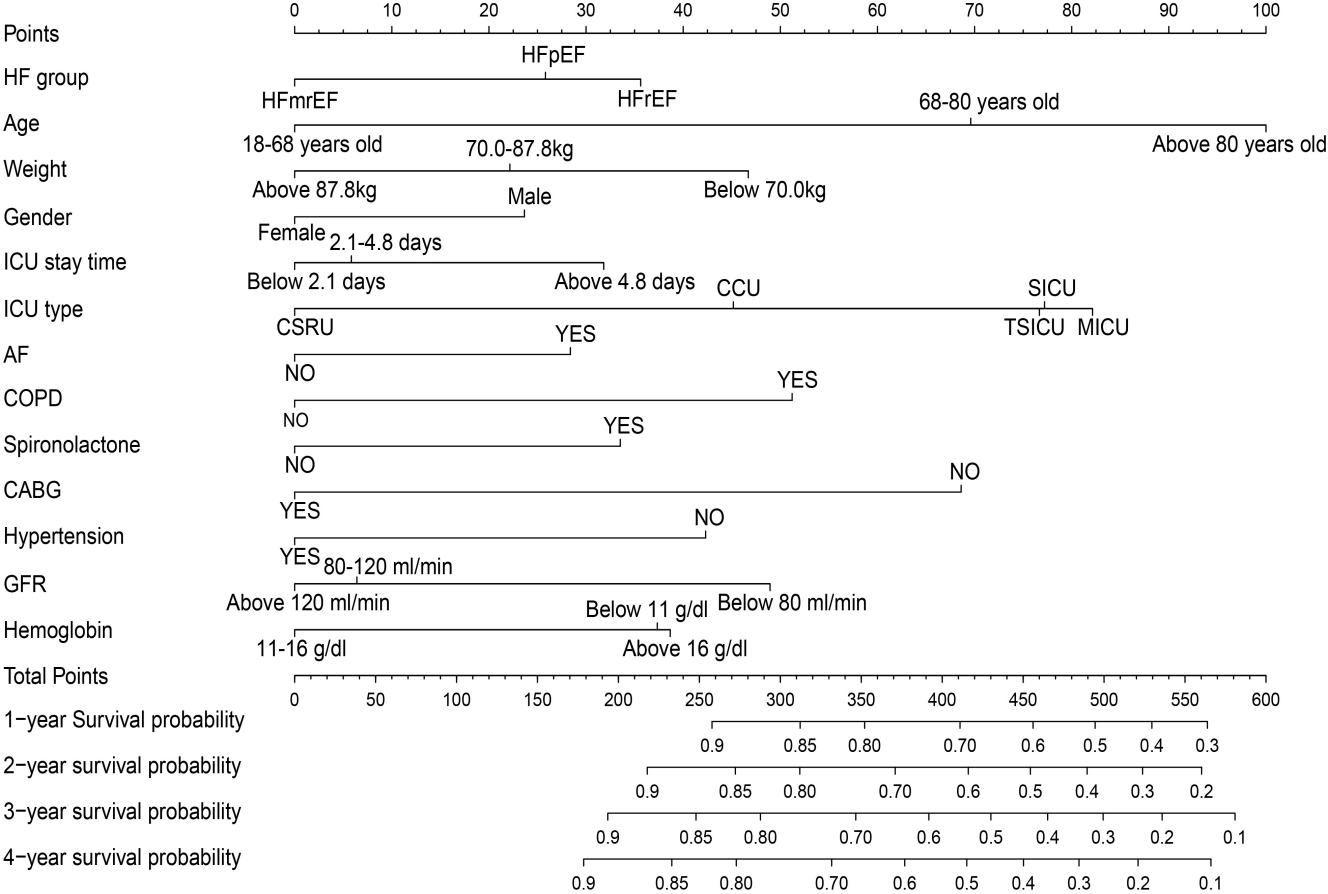
**Nomogram model predict the 1–4 years survival in patients with heart failure**. The nomogram was used by summing all points identified on the scale for each variable. The total points projected on the bottom scales indicate the probabilities of 1–4 years survival.

**Fig. 3.**
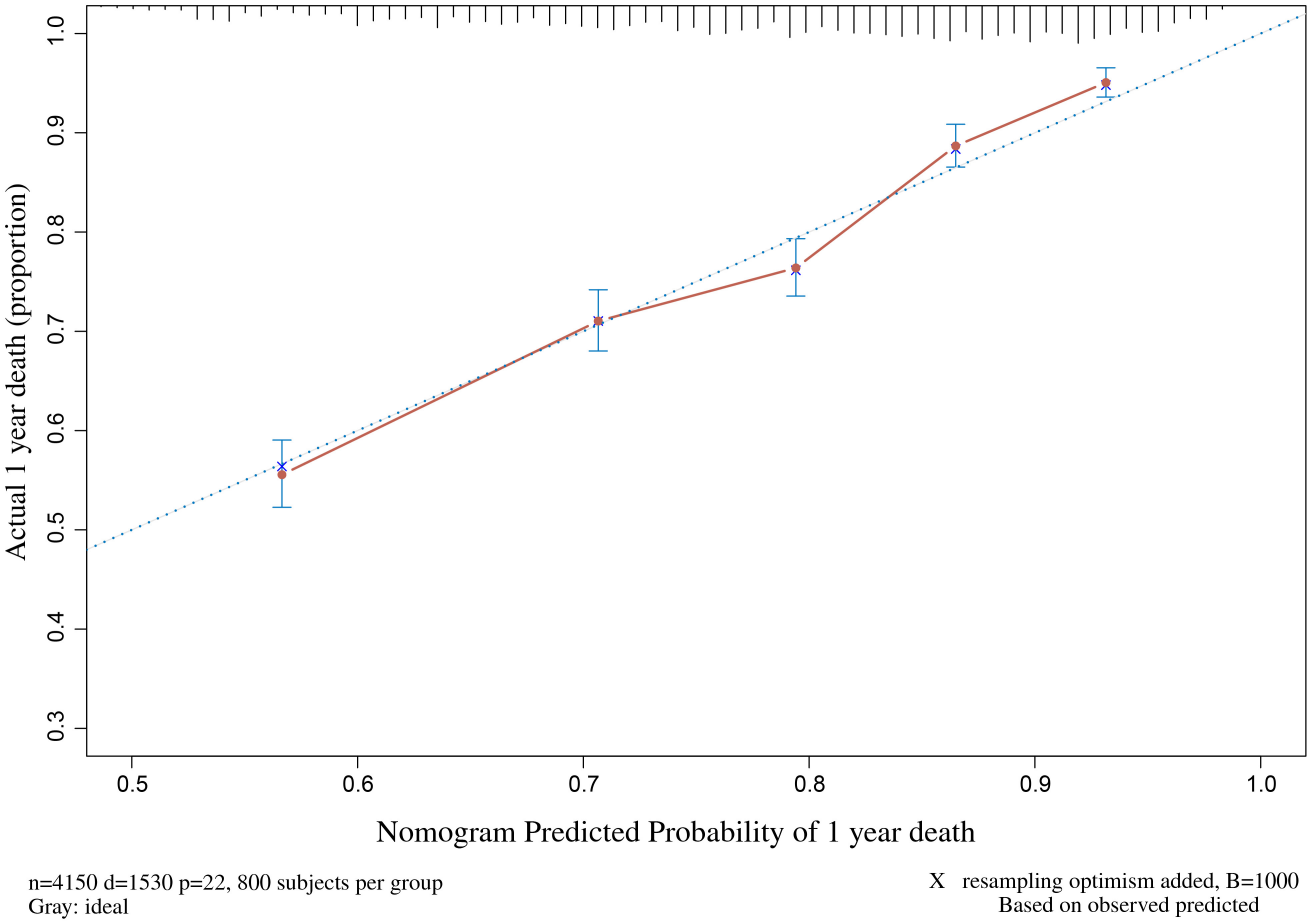
**The calibration curve of the nomogram of 1 year**.

There are a large number of main diagnoses in ICU patients (over 400 types), while ICU types can to a large extent reflect the main diagnoses of patients. We only retain “ICU types” and rather than “main diagnoses” to build a prognostic model, which can increase the clinical utility and simplicity of the model. A novel prognostic prediction model was developed based on intuitive illustration of the 13 independent factors mentioned above. The spline function showed that only the age variable exhibited a linear relationship with prognosis, therefore, patient age was directly involved in model building as a continuous variable (**Supplementary Figs. 2–6**). The model (Fig. 2, **Supplementary Table 3**) demonstrated good discriminative power to estimate life expectancy of HF patients in the ICU with a C-index of 0.70 (95% CI 0.67–0.73), and stable performance in internal validation with a bootstrap-corrected C-index of 0.69. The calibration plot using the all-cause mortality probability of 1 year (Fig. 3) demonstrated a high coherence between the actual observation and predicted values (Fig. 3 and **Supplementary Figs. 7–9**).

### 3.4 Subgroup Analysis

When HFmrEF *vs.* HFpEF was compared, HFmrEF patients in subgroups of age over 80 years old, weight below 70.0 kg, CSRU admission, CABG history, no COPD, GFR below 80 mL/min, and a hemoglobin level below 11 g/dL had significantly better prognosis than HFpEF patients (**Supplementary Fig. 10**). When HFmrEF *vs.* HFrEF was compared HFmrEF patients in subgroups of age over 80 years old, weight below 70.0 kg, male, ICU stay time below 2.1 days, coronary care unit (CCU) or cardiovascular surgery rehabilitation unit (CSRU) admission, hypertension history, no AF and COPD, no spironolactone application, GFR below 80 mL/min, and a hemoglobin level below 11 g/dL had significant better prognosis than HFrEF patients (**Supplementary Fig. 11**). When a comparison of HFrEF *vs.* HFpEF was conducted the prognosis for HFpEF patients with a body weight over 87.8 kg significantly outperformed HFrEF patients, but underperformed the subgroup with a history of CABG (**Supplementary Fig. 12**).

## 4. Discussion

This is a retrospective cohort study aimed to investigate the characteristics and prognosis of HF patients in the ICU with different LVEF values. There was a significant heterogeneity in characteristics among the three HF-groups. Of note, patients with HFmrEF showed superiority in prognosis. We also developed a novel prognostic model specifically for HF patients in ICUs.

### 4.1 Clinical Characteristics among Patients of Different HF-Groups

The proportions of HFpEF patients in this study was 62.75%, which was slightly higher than the numbers found in literature review which ranged from 16% to 62% [7]. However, in other studies, the reported proportion of HFpEF patients was similar, including 64.1% from a Spanish study [8] and 61.90% from a Japanese study [9]. A recent review indicated that the number of HFpEF patients had increased over the past decade [10]. One possible reason for this finding is that the refinement of guidelines has improved the diagnostic strategies used in HF. In addition, there are differing opinions concerning the diagnostic criteria used for HF subtypes, and this directly affects the inclusion criteria and proportions of HFpEF patients described in the literature. Sources of the study samples also have an effect on data obtained, for example, ICU patients were examined in this study, and we included patients who were admitted for other reasons but also suffered from HF and this increased the proportion of patients grouped as HFpEF. Based on the different characteristics of HF subgroups, HFpEF had the highest rate of medical intensive care unit (MICU) admission (42.01%), and HFmrEF CSRU (37.50%), HFrEF CCU (41.25%). Patients with HFpEF were more likely to have COPD, had the highest median age, the highest proportion of females, and the lowest HB and GFR values. Moreover, non-cardiovascular comorbidities in these patients are likely the primary reason for admission to the MICU, which provides better multi-system support and management. Further, these findings were consistent with a study performed by Cheng *et al*. [11]. In this report, HFmrEF patients diverged from HFpEF patients in regard to characteristics germane to the proportion of AMI, coronary disease, hypertension, as well as CABG and percutaneous coronary intervention (PCI) treatments, but were similar to HFrEF patients in these variables. These characteristics indicate a more complicated cardiovascular system disease status and serve to shed light on the high proportion of admissions to the CSRU or CCU among HFmrEF and HFrEF patients, respectively. Moreover, these findings are similar to data provided in previous studies [12]. In terms of main diagnosis, HFrEF patients had the highest proportion of hospitalization due to heart failure, while HFmrEF patients had the highest proportion of myocardial infarction and heart valve disease. HFpEF patients had the highest proportion of septicemia, pneumonia, and other diagnoses among the three types, which was similar to previous studies [1].

A relatively poor renal function and end-stage HF in HFrEF patients usually mean that CABG surgery is less likely to be performed, thus, HFrEF patients were admitted more frequently to CCU but HFmrEF patients were more commonly admitted to the CSRU. Furthermore, HFrEF patients had the longest median ICU stay time (3.10 days), while that of HFmrEF patients was 2.79 days because PCI and CABG were generally short-term or emergency procedures.

Significant discrepancies were observed in renal function, osmotic pressure, white blood cells, and platelets in HFmrEF patients compared to the other HF subtypes. These findings may reflect differences in etiology and pathophysiology among the three HF groups [13]. The decrease in cardiac function observed in HFmrEF and HFrEF patients was predominantly the result of severe cardiovascular diseases such as AMI, myocardial infarction (MI), and coronary heart disease [14]. In contrast, in HFpEF patients’ dysfunction was likely secondary to coordinated development and a combined effect of multi-system disease [15]. With respect to pharmacological treatments in this study, drugs primarily used for non-cardiovascular disease received more attention in HFpEF patients, while there was less emphasis on cardiovascular system drugs, including angiotensin-converting enzyme inhibitors/angiotensin receptor blocker (ACEI/ARB), β-blockers, statins, anticoagulants, aspirin, and adenosine diphosphate (ADP) receptor inhibitors. The distinctions mentioned above, provided a better understanding of current controversy [1, 16] in the clinical management of HFpEF patients.

### 4.2 Prognosis and Its Influence Factors

The 1-year, all-cause mortality rate in this study population was 22.45% in HFrEF patients, 16.35% in HFmrEF patients, and 23.5% in HFpEF patients (*p <* 0.001), and the 4-year mortality rate was 34.55%, 25.00%, and 39.40%, respectively (*p <* 0.001). Taken together, HFmrEF patients presented a favorable prognosis both in the short and long-terms, which was consistent with the results obtained in previous studies [12, 17]. Forest plots indicated specific groups display a prognostic advantage when pairwise comparisons are conducted. For example, HFmrEF patients had a significantly better prognosis compared with both HFpEF and HFrEF patients without COPD complications, but such an advantage was insignificant in patients with COPD. Thus, we propose that HFmrEF patients pay close attention to preventing or alleviating COPD such as taking precautions to prevent chronic bronchitis from developing into COPD. Despite such findings, forest plots could not indicate causal relationships. We observed that the prognosis was similar between HFrEF and HFpEF patients in the first year, but after that HFrEF patients displayed a more favorable prognosis than HFpEF patients (Fig. 1). Based on such findings, we consider that the development of comorbidities and a higher median age may be associated with the Kaplan-Meier curve of HFpEF patients during the following years [12].

Additionally, both Cox univariate and multivariate analyses revealed 14 characteristics including HF subtype, ICU type, ICU stay time, age, weight, gender, hypertension, AF, COPD, CABG, GFR, spironolactone, HB and main diagnoses may potentially serve as independent prognostic factors for all-cause HF mortality. Patients may encounter higher rates of death due to poor laboratory results, ineffective treatment, as well as multiple comorbidities (Table 3). Most of these factors have previously been reported in other studies [18, 19, 20, 21] with consistent effects, except for the presence of hypertension and the application of spironolactone. In this study we found that hypertension was favorable to the prognosis of HF, while in most of the other studies, it was a suggested risk factor [19, 22]. The key to understanding this may be a higher proportion of ACEI/ARB application (65.1%) in hypertensive patients compared to non-hypertensive patients (49.5%, *p <* 0.001). It is well-known that the antihypertensive agents, such as ACEI/ARB, can significantly improve the prognosis of HF by modulating vasodilation, afterload, ventricular remodeling, and neuro-hormonal secretion [1, 23]. HF patients with hypertension may also benefit from earlier and/or larger doses of ACEI/ARB. Patients with main diagnosis of myocardial infarction had a poor prognosis, which we analyzed to be due to the symptoms and signs of heart failure present in the study population. Therefore, further research should be conducted to optimize treatment for patients with myocardial infarction and concomitant heart failure [12].

### 4.3 Spironolactone Application should be More Cautious

Spironolactone has been reported to not significantly reduce the incidence or outcome of cardiovascular-related death or hospitalization in HFpEF and HFmrEF patients [24, 25]; however, spironolactone obviously improved the prognosis of HFrEF [1]. In this study, only 307 patients (7.4%) received spironolactone treatment, which was similar to that reported by Cheng *et al*. [11], but much lower compared to other studies [26, 27]. HFpEF and HFmrEF accounted for 50.16% of the 307 patients, while HFrEF patients accounted for 49.84%. Given guideline recommendations [1] and information gleaned from the literature, we consider that the 50.16% patients might not have achieved satisfactory results after using spironolactone. Forest plots illustrated the prognostic pairwise comparisons among patients who used spironolactone and showed no significant difference in HFpEF and HFmrEF compared with HFrEF. In contrast, in groups without spironolactone treatment, the prognosis of HFmrEF patients was significantly better than that of HFrEF (HR 0.73, 95% CI 0.57–0.94, *p = *0.013, **Supplementary Fig. 11**). A similar trend was also seen in HFpEF *vs.* HFrEF patients although the results were not statistically significant (HR 0.91, 95% CI 0.80–1.03, *p = *0.144, **Supplementary Fig. 12**). The controversial application of spironolactone resulted in it being an independent risk factor for the prognosis of HF (HR 1.33, 95% CI 1.10–1.60, *p = *0.003) in our Cox multivariate analysis. We inferred that spironolactone would not improve the prognosis of ICU HFpEF and HFmrEF patients since such patients were more vulnerable to hypotension, internal environment disorders, or liver and kidney dysfunction [28]. As for HFrEF patients, the suggestion was positive because of its potential beneficial efficacy to the cardiovascular system.

### 4.4 Prognostic Model

Researchers have, in the past, contributed several valid prognostic models for HF patients. The Seattle Heart Failure Model (SHFM), MAGGIC-HF 3A3B score, BCN-Bio-HF [29] and Sheng Jing Heart Failure score [30] are some of the reported model systems. SHFM and BCN-Bio-HF achieved high C-index in the 4th year, being 0.74 (95% CI 0.71–0.77) and 0.77 (95% CI 0.75–0.80), respectively. However, no scoring system serves HF patients in the ICU particularly well. Therefore, our model was developed and quantified intuitively in a nomogram (Fig. 2, **Supplementary Table 3**). The C-index of this model was 0.70 (95% CI 0.67–0.73), which was close to the models outlined above. In addition, the thirteen independent prognostic factors in this model were clinically easy to obtain or measure. Considering the special study populations and the good agreement in internal validation, the model possessed a satisfying predictive effect, and the first-year prediction performance was optimal in this study.

### 4.5 Limitations

This is a retrospective observational cohort study that does present certain limitations. The single-center, retrospective nature of this study may potentially introduce time and regional biases. Physical examination information as well as the B-type natriuretic peptide (BNP) or N-terminal pro-BNP (NT-proBNP) were not analyzed due to a large number of missing data points. However, as an additional diagnostic criterion for HF, the absence of BNP or NT-proBNP did not influence the diagnosis of HF in our study. The predictive model in this study was not validated by external test sets, but this could be accomplished in a further study given a suitable population sample.

## 5. Conclusions

Clinical characteristics and prognosis were significantly different among ICU patients in regards to HFpEF, HFmrEF and HFrEF. Data obtained indicate that HFmrEF patients presented a favorable prognosis in both the short and long-term with lower all-cause mortality rates. Differentiated management strategies for these three subtypes are necessary in clinical work, including complication control, selection and application of specific drugs. Patient features and in-hospital management factors such as age and total ICU stay time independently influenced prognosis of HF patients. The novel prediction model tailored to ICU HF patients showed objective prediction capability.

## Data Availability

The MIMIC-III data used to support the findings of this study may be released upon application to the Medical Information Mart for Intensive Care, who can be contacted at https://mimic.mit.edu/.

## Supplementary Material

Supplementary material associated with this article can be found, in the online version, at https://doi.org/10.31083/j.rcm2406165.
