# Supplementary material for: Characteristics, Prognosis, and Prediction Model of Heart Failure Patients in Intensive Care Units Based on Preserved, Mildly Reduced, and Reduced Ejection Fraction
Source: Rev Cardiovasc Med. 2023 Jun 6;24(6):165. doi: 10.31083/j.rcm2406165 (PMC11264160; doi:10.31083/j.rcm2406165)
Supplement: Supplementary file 1 [file 2153-8174-24-6-165-s1.zip › 2153-8174-24-6-165-s1/Supplementary Figures.docx]

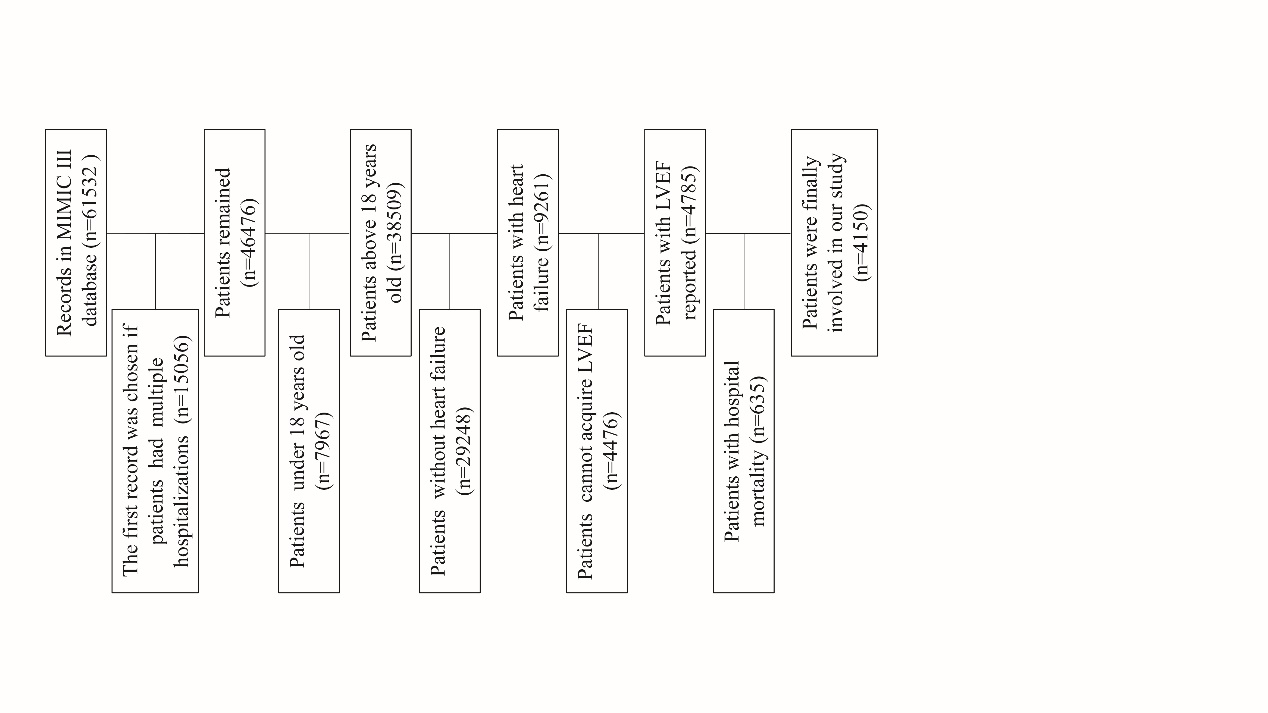


**Supplementary Fig. 1**. Study population.


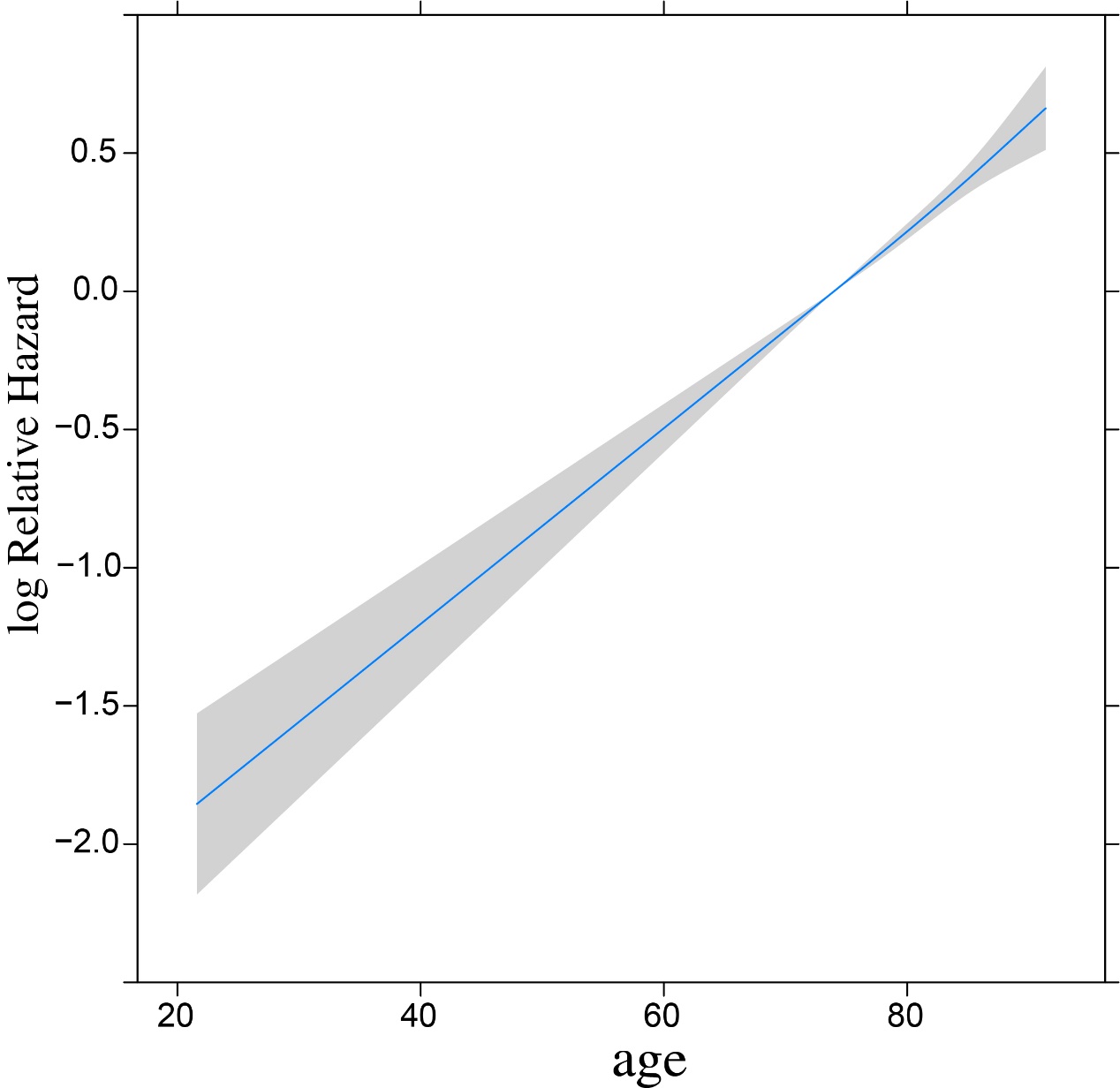


**Supplementary Fig. 2**. The spline curve of age.


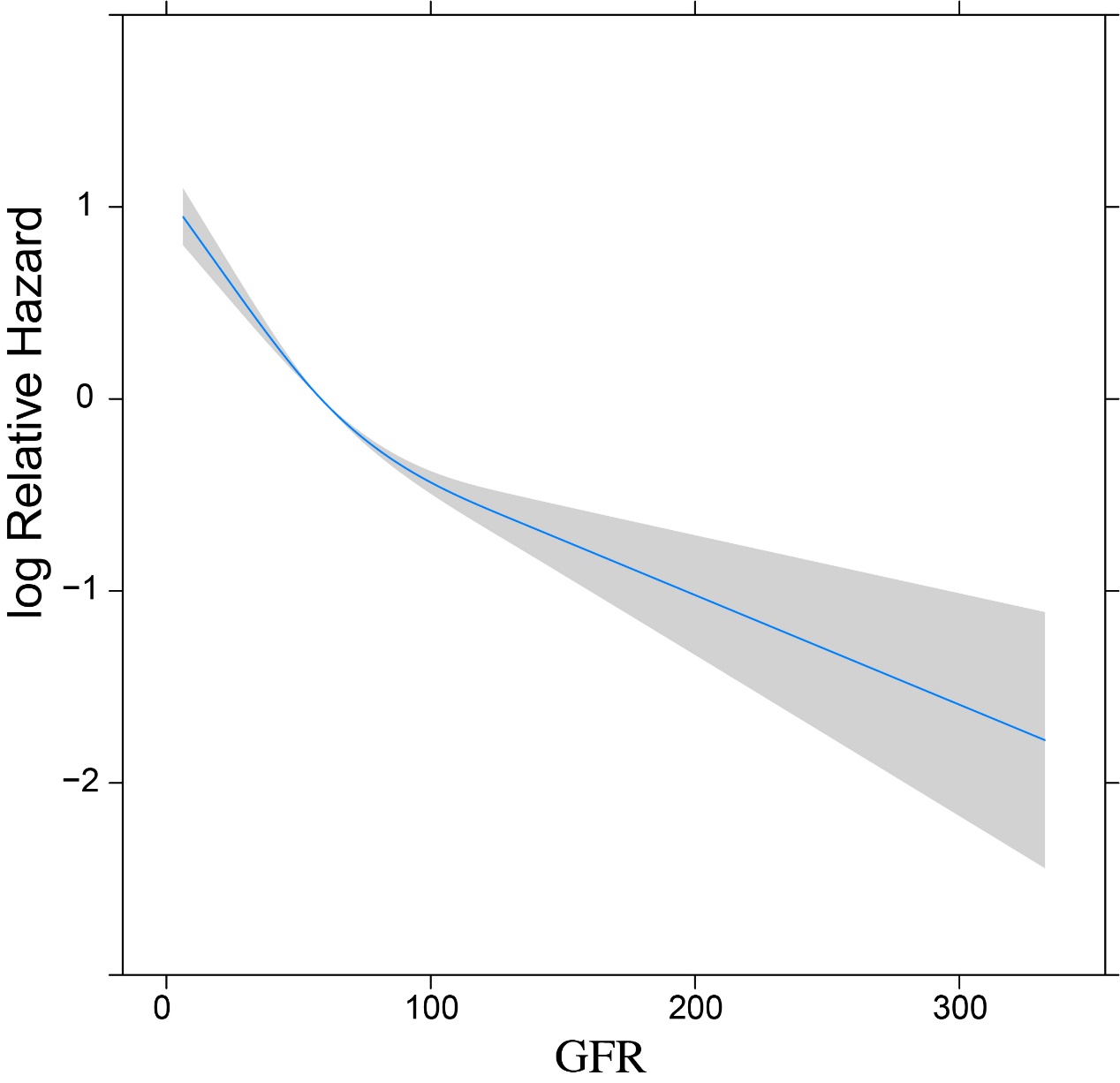


**Supplementary Fig. 3**. The spline curve of GFR.


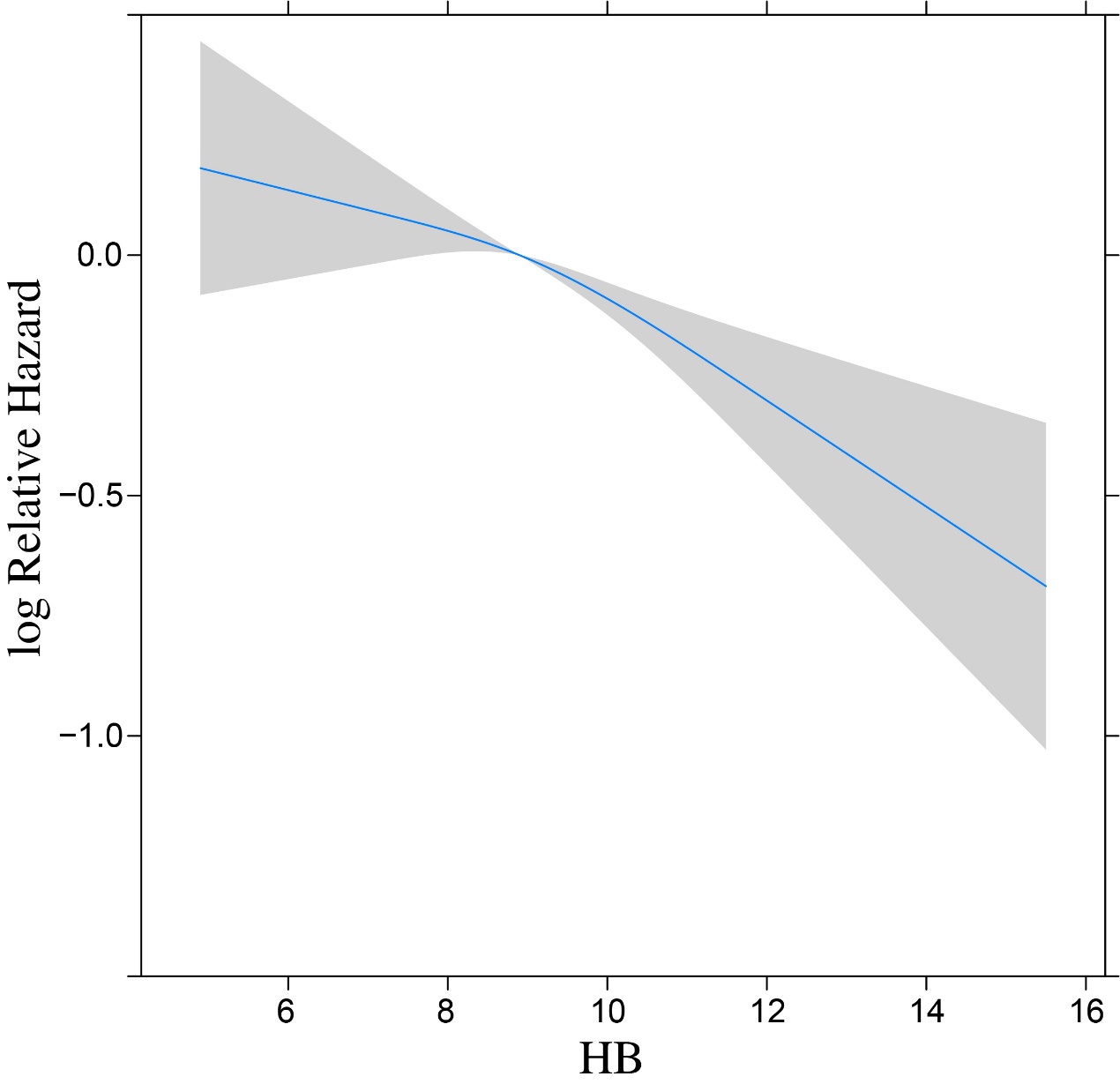


**Supplementary Fig. 4**. The spline curve of HB.


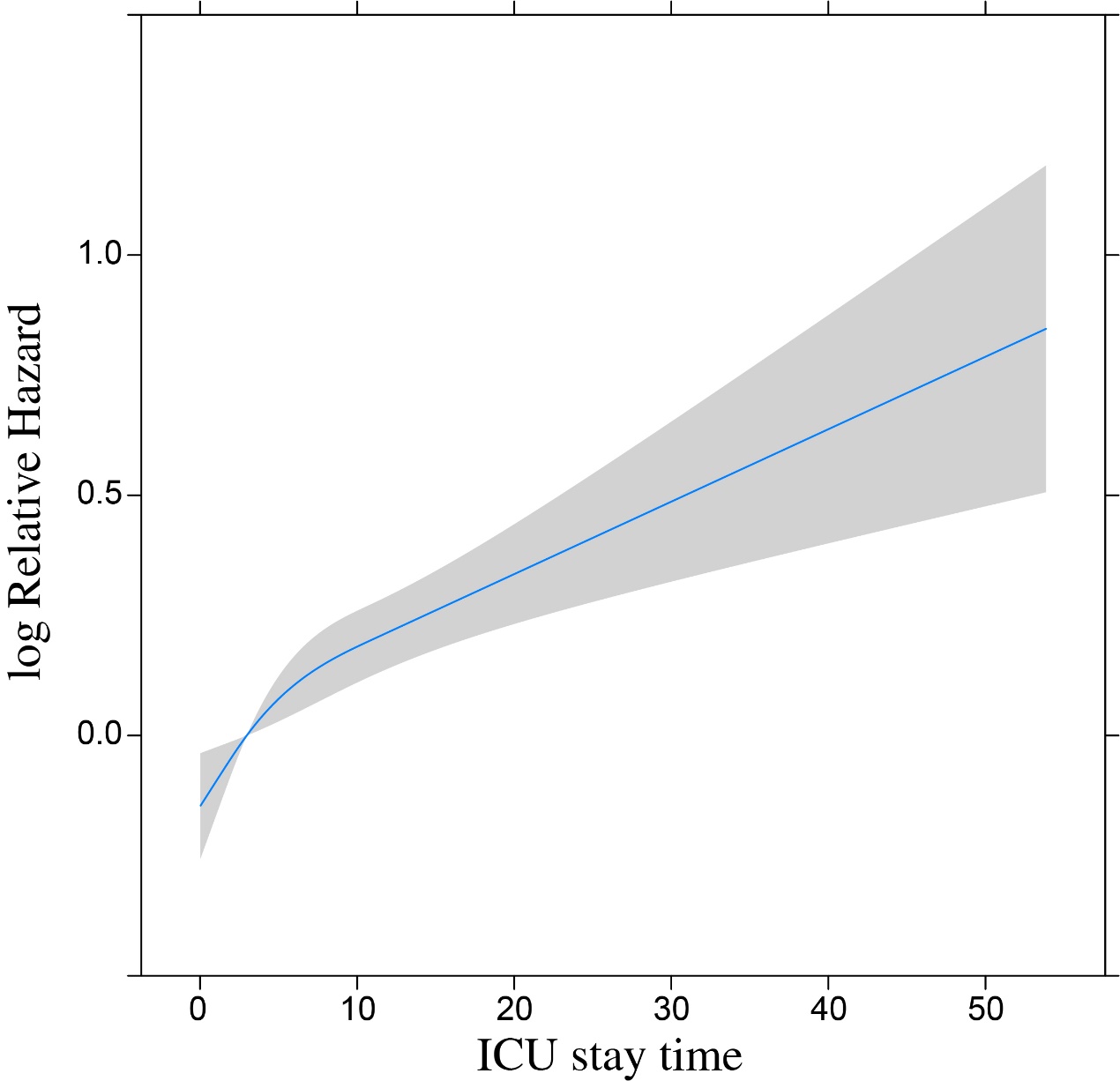


**Supplementary Fig. 5**. The spline curve of ICU stay-time.


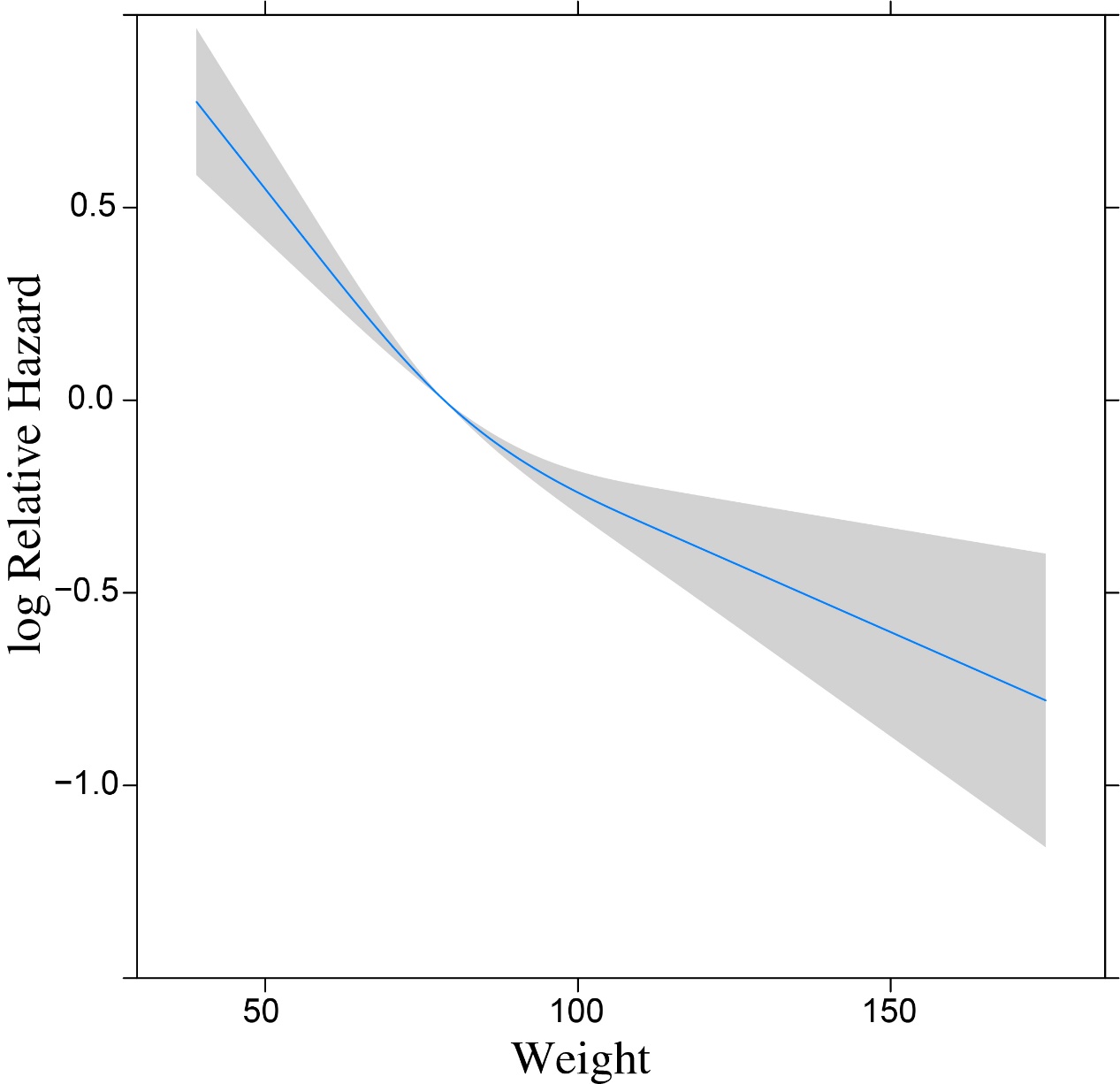


**Supplementary Fig. 6**. The spline curve of weight.


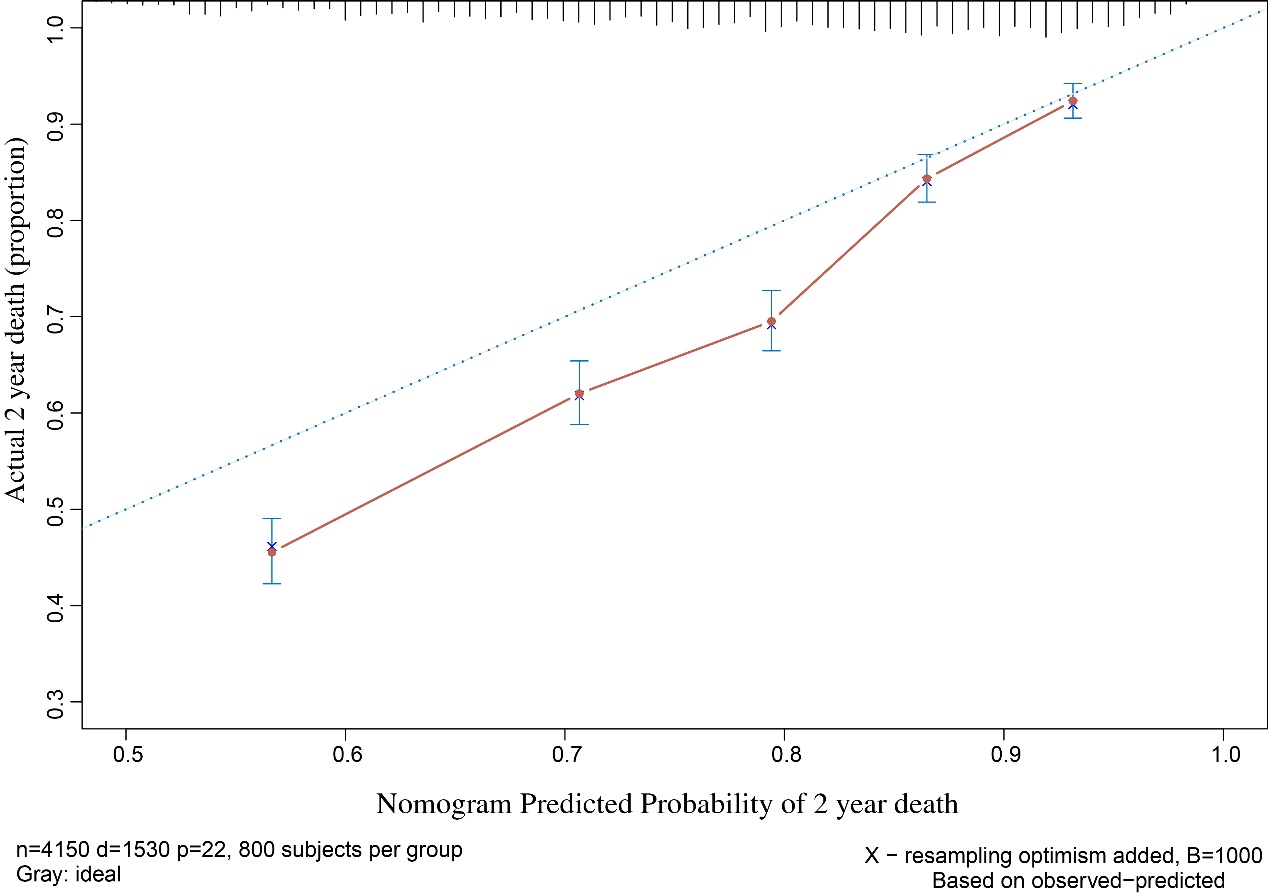


**Supplementary Fig. 7**. The calibration curve of the nomogram of 2 year.


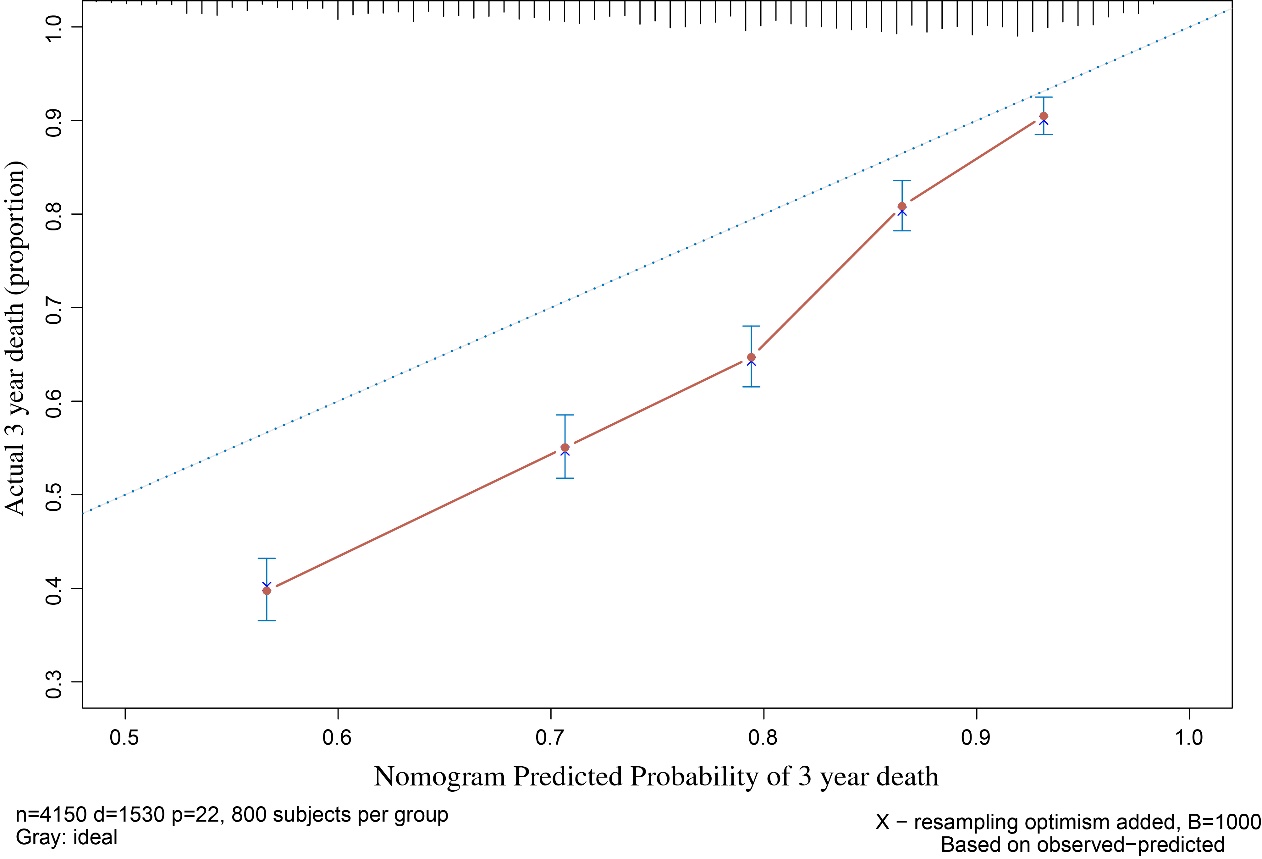


**Supplementary Fig. 8**. The calibration curve of the nomogram of 3 year.


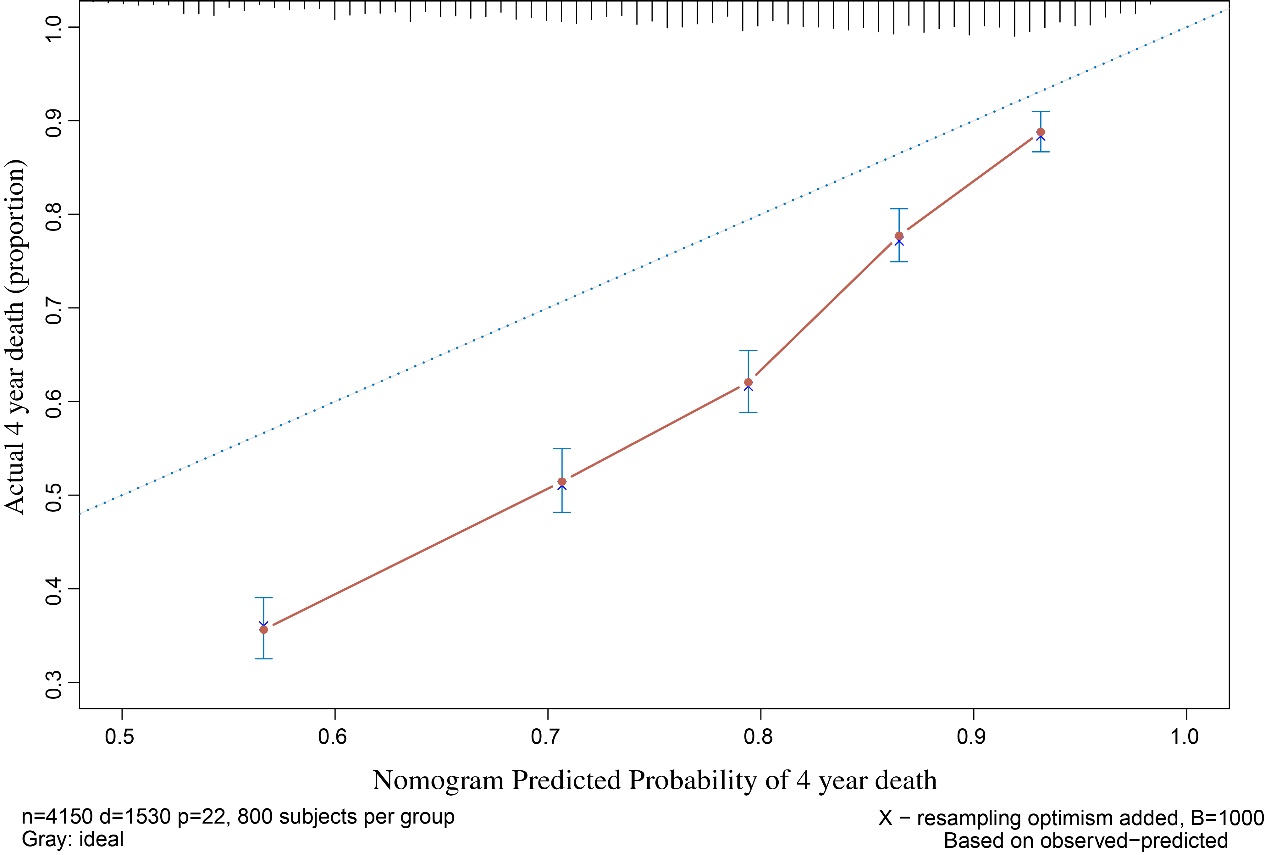


**Supplementary Fig. 9**. The calibration curve of the nomogram of 4 year.


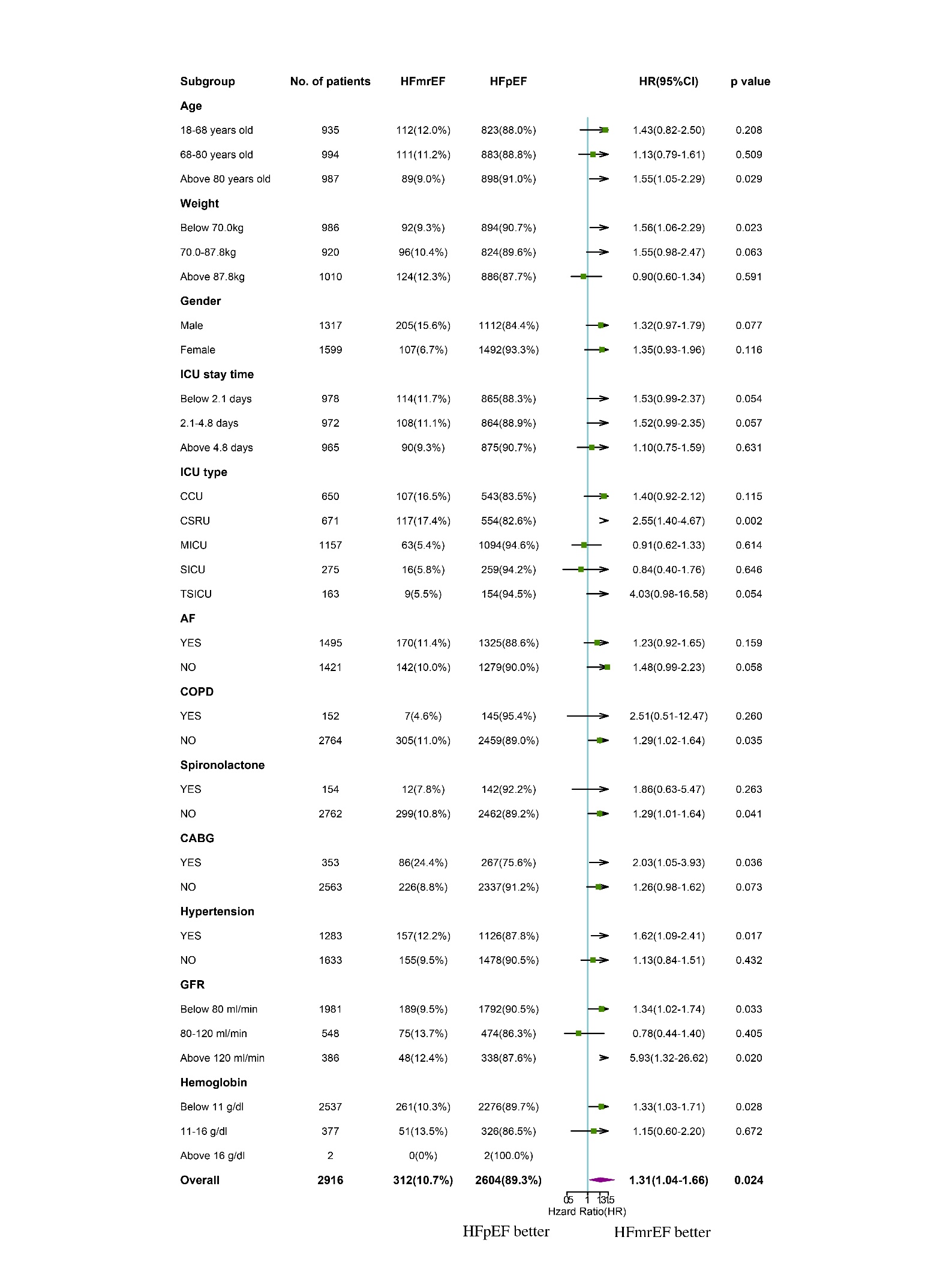


**Supplementary Fig. 10**. Forest plot of heart failure with mildly reduced ejection fraction (HFmrEF) *vs.* heart failure with preserved ejection fraction (HFpEF).


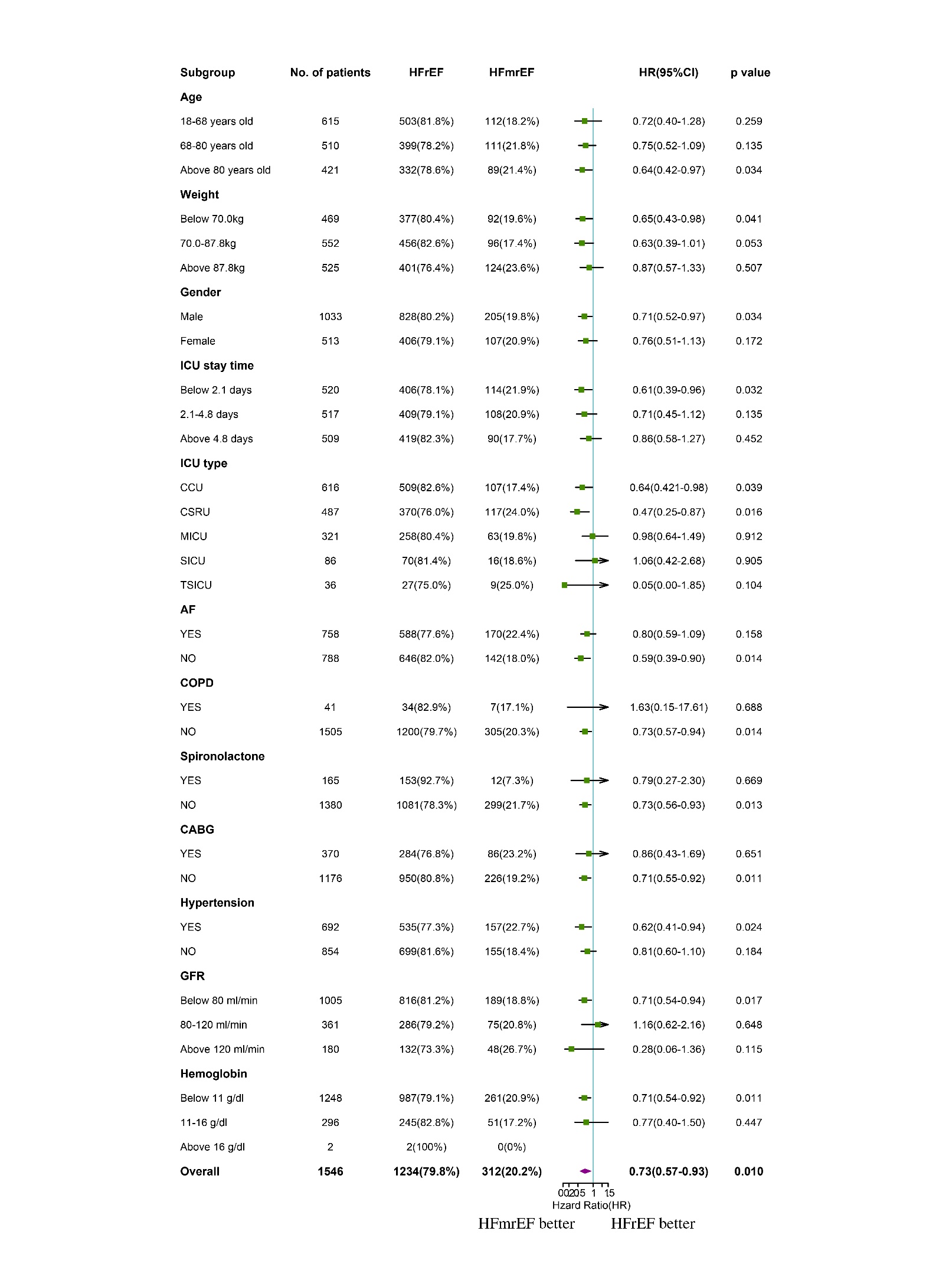


**Supplementary Fig. 11**. Forest plot of heart failure with reduced ejection fraction (HFrEF) *vs.* HFmrEF.


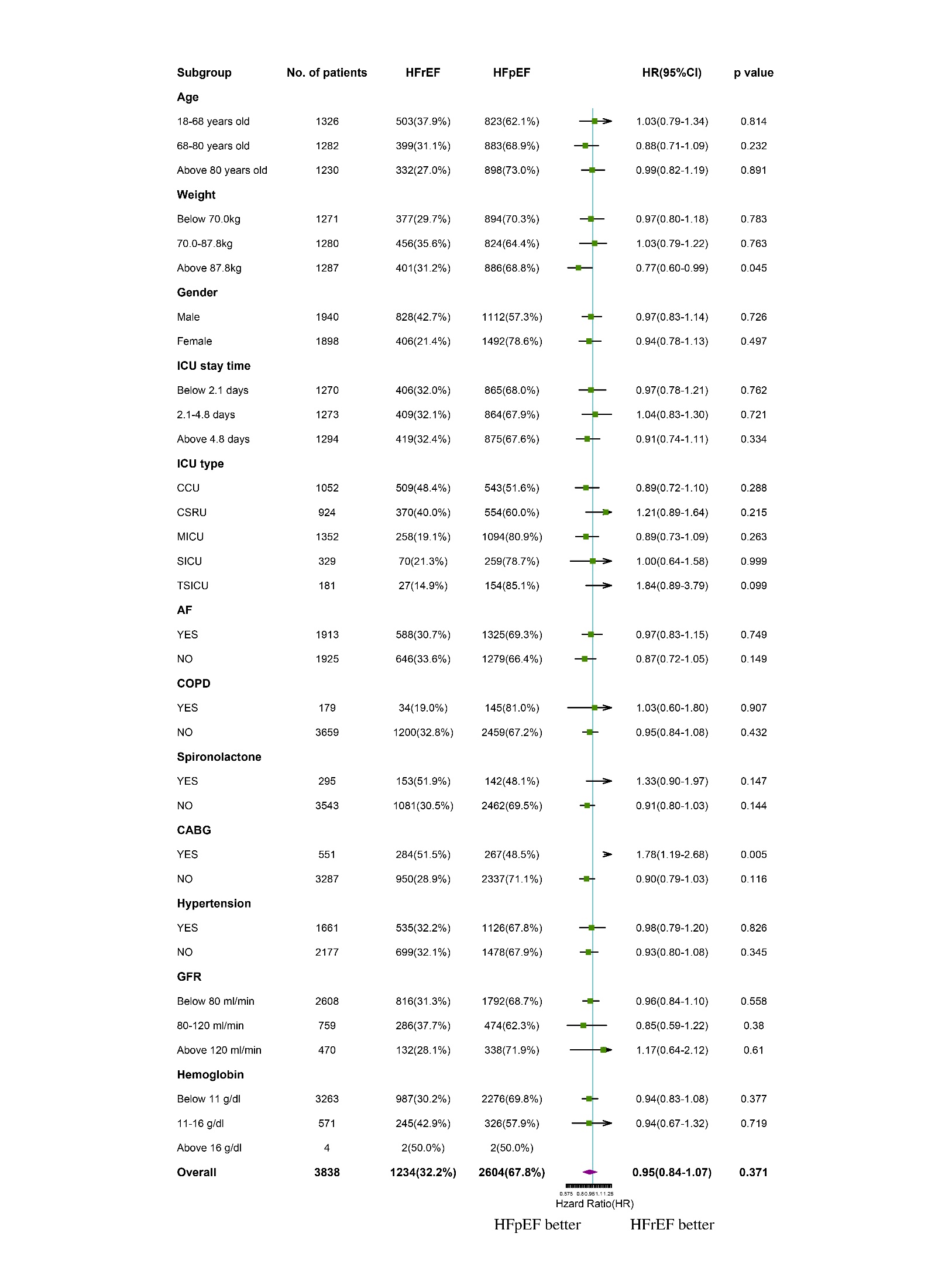


**Supplementary Fig. 12**. Forest plot of HFrEF *vs.* HFpEF.
