# Supplementary material for: Characteristics, Prognosis, and Prediction Model of Heart Failure Patients in Intensive Care Units Based on Preserved, Mildly Reduced, and Reduced Ejection Fraction
Source: Rev Cardiovasc Med. 2023 Jun 6;24(6):165. doi: 10.31083/j.rcm2406165 (PMC11264160; doi:10.31083/j.rcm2406165)
Supplement: Supplementary file 1 [file 2153-8174-24-6-165-s1.zip › 2153-8174-24-6-165-s1/Supplementary Tables.DOCX]

**Supplementary Table 1**. 4 years all-cause mortality after discharge.

|  | HFrEF  (n=1234, 29.73%) | HFmrEF  (n=312, 7.50%) | HFpEF  (n=2604, 62.75%) | Overall  *p* | HFrEF *vs.* HFmrEF *p* | HFmrEF *vs.* HFpEF *p* |
| --- | --- | --- | --- | --- | --- | --- |
| First year | 277 (22.45) | 51 (16.35) | 612 (23.50) | <0.001 | 0.400 | 0.004 |
| Second year | 76 (6.16) | 10 (3.21) | 187 (7.18) | 0.022 | 0.042 | 0.008 |
| Third year | 43 (3.84) | 14 (4.49) | 134 (5.15) | 0.072 | 0.401 | 0.616 |
| Fourth year | 27 (2.19) | 2 (0.64) | 93 (3.57) | 0.002 | 0.072 | 0.006 |
| Four years | 426 (34.5) | 78 (25.00) | 1026 (39.40) | <0.001 | 0.001 | <0.001 |

**Supplementary Table 2**. Univariate Cox proportional-hazards regression model for all-cause mortality.

|  | HR (95% CI) | *P* |
| --- | --- | --- |
| HF-group |  |  |
| HFrEF | REF | REF |
| HFmrEF | 0.68 (0.53, 0.86) | 0.002 |
| HFpEF | 1.16 (1.04, 1.30) | 0.010 |
| Age (per 1 year) | 1.04 (1.03, 1.04) | <0.001 |
| Weight (kg) |  |  |
| <70 | REF | REF |
| 70–87.8 | 0.74 (0.66, 0.84) | <0.001 |
| >87.8 | 0.52 (0.46, 0.59) | <0.001 |
| Gender |  |  |
| Female | REF | REF |
| Male | 0.90 (0.82, 1.00) | 0.045 |
| ICU stay time(days) |  |  |
| <2.1 | REF | REF |
| 2.1–4.8 | 1.08 (0.95, 1.23) | 0.228 |
| >4.8 | 1.31 (1.16, 1.48) | <0.001 |
| ICU type |  |  |
| CCU | REF | REF |
| CSRU | 0.54 (0.45, 0.63) | <0.001 |
| MICU | 1.46 (1.29, 1.66) | <0.001 |
| SICU | 1.34 (1.12, 1.62) | 0.002 |
| TSICU | 1.47 (1.17, 1.84) | 0.001 |
| SOFA first |  |  |
| 0–3 | REF | REF |
| 3–6 | 1.16 (1.03, 1.30) | 0.013 |
| >6 | 1.12 (0.98, 1.27) | 0.090 |
| AMI | 0.66 (0.52, 0.84) | 0.001 |
| MI | 0.79 (0.69, 0.92) | 0.002 |
| AF | 1.47 (1.33, 1.63) | <0.001 |
| Coronary disease | 0.79 (0.71, 0.87) | <0.001 |
| COPD | 1.88 (1.54, 2.29) | <0.001 |
| Hypertension | 0.66 (0.59, 0.73) | <0.001 |
| Diabetes | 0.88 (0.79, 0.98) | 0.017 |
| CABG | 0.38 (0.31, 0.46) | <0.001 |
| PCI | 0.83 (0.69, 1.01) | 0.067 |
| ACEI/ARB | 0.77 (0.70, 0.85) | <0.001 |
| β-blocker | 0.82 (0.72, 0.94) | 0.003 |
| Loop diuretics | 1.04 (0.90, 1.21) | 0.578 |
| Spironolactone | 1.29 (1.08, 1.54) | 0.005 |
| Statin | 0.80 (0.72, 0.89) | <0.001 |
| Anticoagulant | 1.02 (0.92, 1.13) | 0.667 |
| Aspirin | 0.75 (0.67, 0.84) | <0.001 |
| Anti-ADP | 0.94 (0.83, 1.06) | 0.275 |
| Digitalis | 1.38 (1.18, 1.61) | <0.001 |
| GFR (mL/min) |  |  |
| <80 | REF | REF |
| 80–120 | 0.42 (0.36, 0.49) | <0.001 |
| >120 | 0.33 (0.27, 0.41) | <0.001 |
| HB min (g/dL) |  |  |
| <11 | REF | REF |
| 11–16 | 0.73 (0.63, 0.85) | <0.001 |
| >16 | 0.65 (0.09, 4.59) | 0.662 |
| K max (mmol/L) |  |  |
| <3.5 | REF | REF |
| 3.5–5.5 | 0.68 (0.31, 1.52) | 0.346 |
| >5.5 | 0.62 (0.28, 1.40) | 0.250 |
| K min (mmol/L) |  |  |
| <3.5 | REF | REF |
| 3.5–5.5 | 0.88 (0.80, 0.98) | 0.020 |
| >5.5 | NON |  |
| WBC max (10^9^/L) |  |  |
| <3.5 | REF | REF |
| 3.5–9.5 | 0.70 (0.37, 1.32) | 0.269 |
| >9.5 | 0.60 (0.32, 1.11) | 0.105 |
| POP (mmol/L) |  |  |
| <280 | REF | REF |
| 280–320 | 0.94 (0.72, 1.21) | 0.611 |
| >320 | 1.69 (1.25, 2.30) | 0.001 |
| Main diagnoses |  |  |
| MI | REF | REF |
| HF | 0.61 (0.53, 0.71) | <0.001 |
| Cardiacvalve disease | 1.03 (0.88, 1.20) | 0.714 |
| Septicemia | 0.31 (0.24, 0.39) | <0.001 |
| Hemorrhage | 1.13 (0.93, 1.36) | 0.218 |
| Respiratory failure | 1.18 (0.95, 1.47) | 0.144 |
| Pneumonia | 1.03 (0.81, 1.31) | 0.818 |
| Acute kidney failure | 1.06 (0.78, 1.43) | 0.708 |
| others | 1.43 (1.04, 1.96) | 0.029 |

**Supplementary Table 3**. Nomograph scoring details.

| Variable | Score | Variable | Score | Variable | Score |
| --- | --- | --- | --- | --- | --- |
| **HF-group** |  | **Age** |  | **Weight** |  |
| HFrEF | 13 | 10 | 0 | <70 | 15 |
| HFmrEF | 0 | 20 | 11 | 70–87.8 | 7 |
| HFpEF | 10 | 30 | 100 | >87.8 | 0 |
|  |  | 40 | 33 |  |  |
|  |  | 50 | 44 |  |  |
|  |  | 60 | 56 |  |  |
|  |  | 70 | 67 |  |  |
|  |  | 80 | 78 |  |  |
|  |  | 90 | 89 |  |  |
|  |  | 100 | 100 |  |  |
| **Gender** |  | **ICU stay time** |  | **ICU type** |  |
| Male | 8 | <2.1 | 0 | CCU | 14 |
| Female | 0 | 2.1–4.8 | 2 | CSRU | 0 |
|  |  | >4.8 | 11 | MICU | 26 |
|  |  |  |  | SICU/TSICU | 24 |
| **AF** |  | **COPD** |  | **Spironolactone** |  |
| YES | 8 | YES | 17 | YES | 12 |
| NO | 0 | NO | 0 | NO | 0 |
| **CABG** |  | **Hemoglobin** |  | **GFR** |  |
| YES | 12 | <11 | 12 | <80 | 12 |
| NO | 0 | 11–16 | 0 | 80–120 | 0 |
|  |  | >16 | 17 | >120 | 1 |
| **Hypertension** |  |  |  |  |  |
| YES | 0 |  |  |  |  |
| NO | 15 |  |  |  |  |
| Total Points 1-year Survival probability  134 0.9  152 0.85  164 0.80  183 0.70  198 0.6  210 0.5  221 0.4  232 0.3  Total Points 2-year survival probability  121 0.9  139 0.85  151 0.80  170 0.70  185 0.6  197 0.5  208 0.4  219 0.3  231 0.2  Total Points 3-year survival probability  113 0.9  131 0.85  144 0.80  163 0.70  177 0.6  189 0.5  200 0.4  211 0.3  223 0.2  238 0.1  Total Points 4-year survival probability  109 0.9  126 0.85  139 0.80  158 0.70  172 0.6  184 0.5  196 0.4  207 0.3  218 0.2  233 0.1 | | | | |  |
